# Virtual Reality Reward Training for Anhedonia: A Pilot Study

**DOI:** 10.3389/fpsyg.2020.613617

**Published:** 2021-01-07

**Authors:** Kelly Chen, Nora Barnes-Horowitz, Michael Treanor, Michael Sun, Katherine S. Young, Michelle G. Craske

**Affiliations:** ^1^Department of Psychology, University of Arizona, Tucson, AZ, United States; ^2^Department of Psychology, University of California, Los Angeles, Los Angeles, CA, United States; ^3^Department of Psychiatry, University of California, Los Angeles, Los Angeles, CA, United States; ^4^Department of Psychology, Dartmouth College, Hanover, NH, United States; ^5^Social, Genetic and Developmental Psychiatry Centre, King’s College London, London, United Kingdom

**Keywords:** anhedonia, depression, virtual reality, emotion, positive affect, treatment

## Abstract

Anhedonia is a risk factor for suicide and poor treatment response in depressed individuals. Most evidence-based psychological therapies target symptoms of heightened negative affect (e.g., negative inferential style) instead of deficits in positive affect (e.g., attenuated reward response) and typically show little benefit for anhedonia. Viewing positive scenes through virtual reality (VR) has been shown to increase positive affect and holds great promise for addressing anhedonic symptoms. In this pilot study, six participants with clinically significant depression completed 13 sessions of exposure to positive scenes in a controlled VR environment. Significant decreases were found in self-reported anhedonia, depression, anxiety, and impairments in functioning from baseline to 1-month follow-up. Negative affect decreased over all 13 sessions, and positive affect increased over sessions 8–13. Results suggest that positive experiences in VR may be a novel avenue for the treatment of anhedonia in depressed individuals.

## Introduction

Anhedonia, the diminished capacity to experience joy or pleasure, is a common symptom across psychiatric diagnoses including depression (Fawcett et al., 1990; Morris et al., 2009), social anxiety (Brown et al., 1998; Kashdan, 2007), schizophrenia (Watson and Naragon-Gainey, 2010), and substance use disorder (American Psychiatric Association, 2013; Thomsen et al., 2015). Moreover, anhedonia decreases treatment response and increases risk for suicide, and standard antidepressant medication treatments may even worsen anhedonic symptoms (Fawcett et al., 1990; Morris et al., 2009; McCabe et al., 2010). Additionally, most evidence-based psychological therapies typically target negative affect and have had relatively limited impact upon anhedonia. The mechanisms of this ineffectiveness have been attributed to a number of factors including reversal of motivational state (Winer and Salem, 2016) as well as fear of positive emotion (Vanderlind et al., 2017). Behavioral activation (BA), a component of cognitive behavioral therapy (CBT) predicated upon principles of positive reinforcement, depends upon the availability of and the motivation to engage in rewarding activities. However, the lack of motivation to pursue such activities presents a significant obstacle. It is therefore unsurprising that BA was also found to be relatively ineffective for anhedonia (Moore et al., 2013). Existing literature has suggested that mental imagery might be more effective than a verbal approach in accessing and modifying emotion in therapy (Holmes et al., 2008), and studies combining mental imagery and BA have resulted in greater behavioral activation than BA alone (Renner et al., 2017). However, it remains unknown whether these results can be extended to anhedonia, which warrants closer examination as a treatment target. Recently developed treatments have begun to investigate methods of targeting deficits in positive affect, but most require implementation by a trained clinician, which could pose difficulties in terms of accessibility (Dunn, 2012; Taylor et al., 2017).

Positive Affect Treatment (PAT) is one such clinician-delivered treatment that directly targets deficits in reward processes underlying anhedonia (Craske et al., 2016, 2019). A major portion of PAT is the combination of BA with imaginal recounting during which the most positive elements of the BA experience are repeatedly recalled and rehearsed in detail for prolonged periods of time. The results from PAT are promising, showing significant improvements in positive affect (Craske et al., 2019). Nonetheless, for some patients, engagement in behaviorally rewarding activities is thwarted by lack of motivation.

Virtual reality (VR) offers a potential solution for such patients. The following VR protocol was developed from the PAT protocol (Craske et al., 2016, 2019) and targets both finding and engaging in rewarding activities as well as savoring pleasurable moments to enhance the hedonic impact of these activities. Virtual reality exposure facilitates this process by circumventing obstacles to traditional behavioral activation (e.g., finding and engaging in rewarding activities) posed by anhedonia.

The positive experiences made possible in VR are immersive, and the virtual contexts are more controlled relative to typical therapeutic settings. This may more robustly engage reward circuitry than typical therapeutic mechanisms. This immersive quality has lent VR to use in exposure treatments for anxiety (Miloff et al., 2019) and other interventions focused on pain management (Kenney and Milling, 2016) and relaxation (Anderson et al., 2017). Positive scenes in VR have been shown to effectively increase positive affect and expose individuals to rewarding activities with minimal effort (Garcia-Palacios et al., 2015).

However, no studies to date have evaluated VR specifically for anhedonia. Thus, the goal of this pilot study was to explore VR as a tool for exposure to positively reinforcing stimuli. We hypothesized that anhedonia in depressed individuals would decrease as a function of exposure to positive VR scenes followed by imaginal recounting.

## Methods

Participants with clinically significant depression were provided VR behavioral activation with imaginal recounting for 13 hour-long sessions over a 7-week period with an average of 3 days between sessions (see Supplementary Figure 1 for a timeline of assessments)^1^. These sessions took place in a private experiment room located in the Anxiety and Depression Research Center within the University of California, Los Angeles (UCLA) campus. Trained research assistants familiar with the Positive Affect Treatment protocol conducted each session. Assessments were conducted at baseline, mid-treatment, post-treatment, and 1-month follow-up.

### Participants

Six participants (five female, *M*_age_ = 24.67 years, SD = 5.68 years) participated in the study (see Table 1 for descriptive statistics). Participants were recruited online through the UCLA Depression Grand Challenge website, and UCLA students were also recruited through flyers placed around campus. Four of the six final participants were undergraduate students at UCLA, and two were community respondents. All participants completed informed consent (UCLA Medical Institutional Review Board 3, IRB#16-001568).

**TABLE 1:** Sample descriptive statistics.

	*M*	SD	Range
*Age (years)*	24.67	5.68	19–32
*Current MDE (months)*	14.67	15.02	1–36
*Number of previous episodes*	2.33	2.25	0–5
*Duration of lifetime illness (years)*	4.71	3.43	0.25–10

Initial eligibility was determined after participants completed an online screening questionnaire. To meet eligibility criteria, participants were between the ages of 18 and 40, fluent in English, either not taking or stable on psychotropic medications for 3 months, agreed to refrain from taking any additional psychotropic medications or psychosocial treatments for the duration of the study, and able to visit the laboratory twice per week for the duration of the study.

After screening, participants completed self-report measures, and eligibility was determined according to the following clinical cutoffs: Behavioral Activation Scale (BAS; Carver and White, 1994) Reward Drive subscale score ≤11, Fun Seeking subscale score of ≤11, or Reward Responsiveness subscale score of ≤16 to indicate low levels of reward sensitivity; Computerized Adaptive Testing-Depression (CAT-DI) score ≥65 to indicate at least moderately high levels of depression symptoms (Gibbons et al., 2012); and Sheehan Disability Scale (SDS) score ≥5 to represent increased risk of mental health-related impairment (Leon et al., 1997). The BAS cutoffs were selected for low levels of reward sensitivity given its relevance to unipolar depression (Alloy et al., 2016). Self-reported levels of depression (CAT-DI) and functional impairment (SDS) were selected in order to target a sufficiently impaired sample for which symptom improvement would be meaningful. An additional cutoff was established for the average visual, auditory, and organic subscale scores of the Questionnaire Upon Mental Imagery (QMI ≤ 3; Burdea and Coiffet, 2003; Iachini et al., 2019). The QMI cutoff was included to select for good mental imagers, which has been shown to correlate with immersion in virtual reality experiences (Iachini et al., 2019). This particular cutoff was determined from the top 10% of scores from a separate, earlier sample of 50 undergraduate students at UCLA.

Participants were excluded if they had metal implants, were claustrophobic, pregnant, left-handed, or experienced frequent motion sickness. Additional exclusion criteria, established during the Structured Clinical Interview for the DSM-5 Research Version (SCID-5; First et al., 2015), were lifetime history of bipolar disorder, psychosis, suicide attempts, intellectual disability or organic brain damage, or current substance use disorder within the last 6 months. SCID-5 interviews were administered by trained and reliability-certified advanced graduate students and research assistants. The exclusion criteria were established based on MRI contraindications (e.g., metal implants, pregnancy, and left-handedness), the results of which are not included in this manuscript. Individuals who experienced frequent motion sickness were also excluded given the increased likelihood of motion sickness in a VR environment (Munafo et al., 2017). SCID-5 diagnoses were included among the exclusion criteria to avoid confounding factors.

### VR Treatment

Each treatment session (13 hour-long sessions across 7 weeks) comprised three stages: VR viewing, imaginal recounting, and homework. All participants completed all sessions.

#### VR Viewing

Each viewing session consisted of a 12–15-min block of VR viewing of two to four scenes on an Oculus Rift CV1. The VR content was selected from the Oculus Library (v.1.33.0.750915) based on ratings of low nausea, positive valence, and high arousal made by trained research assistants. Sessions one through seven consisted of predominantly positively valenced content that was replaced by increasingly more neutral, but not negative, content in sessions eight through 13. This was so participants became trained in recalling the most positive features of the VR viewing even when surrounded by neutral stimuli.

#### Imaginal Recounting

Participants were then prompted to choose one of the VR scenes they had just viewed and write a description including the most positive part of the scene and positive emotions or sensations experienced. Following the written recounting, participants listened to a 6-min guided audio recording instructing them to imaginally recount their chosen VR scene. Each VR imaginal recounting was followed by recounting of an autobiographical memory in order to increase the transfer of VR training effects to personally relevant experience. This is warranted given that depression is associated with: deficits in generating vivid past positive mental images (Werner-Seidler and Moulds, 2011) and overgeneral autobiographical memory (Holmes et al., 2016) or failure to generate specific memories that take place within the span of a single event or day (Williams et al., 2007), which predicts onset and poorer course of depression (Brewin, 2006; Barry et al., 2019; Hallford et al., 2020). Participants wrote a detailed recollection of an autobiographical memory in response to a three-word prompt, including the most positive part as well as positive emotions or sensations experienced. The first seven sessions utilized explicitly positive three-word prompts (e.g., “happy, sweet, and nice”) while the final six sessions used less explicitly positive prompts (e.g., “spring, music, and birthday”). Then, participants listened to a 6-min guided audio recording instructing them to imaginally recall their chosen autobiographical memory.

#### Homework

Participants were instructed to complete the same sequence of imaginal recounting at home with the aid of digital copies of the guided audio recalls. Homework was checked for completion in-session.

### Outcome Measures

#### Anhedonia, Depression, Anxiety, and Functional Impairment

Symptoms were assessed at baseline, mid-treatment, post-treatment, and 1-month follow-up. Cronbach’s alphas are reported for the current sample. Anhedonia symptom severity was measured with the 22-item Anhedonic Depression subscale of the Mood and Anxiety Symptoms Questionnaire (MASQ-AD), which demonstrated excellent internal consistency (α = 0.838) and has demonstrated good convergent and discriminant validity (Watson et al., 1995).

Self-reported depression and anxiety were measured using the Computerized Adaptive Testing (CAT) tool depression inventory (CAT-DI) and anxiety inventory (CAT-ANX; Gibbons et al., 2008, 2012, 2014). The 3-item Sheehan Disability Scale (SDS) was used to measure self-reported level of functional impairment due to mood symptoms. This scale had good internal consistency (α = 0.865) and has demonstrated validity (Sheehan et al., 1996; Arbuckle et al., 2009).

#### Reward Sensitivity

Reward sensitivity was measured using self-report questionnaires at baseline, mid-treatment, post-treatment and 1-month follow-up. Questionnaires included the BAS, which possessed adequate internal consistency (α = 0.767) and has demonstrated validity (Carver and White, 1994; Jorm et al., 1998) and the 18-item Temporal Experience of Pleasure Scale (TEPS), which had adequate internal consistency (α = 0.740) and has demonstrated validity (Garfield et al., 2016). The TEPS has two subscales to measure expectancy of reward (Anticipation) and responsiveness to reward attainment (Consummatory; Gard et al., 2006).

#### Positive and Negative Affect

Before and after each VR session, participants completed the 20-item Positive and Negative Affect Schedule (PANAS; Watson et al., 1988). The scale is divided into two 10-item positive affect (PA) and negative affect (NA) subscales. The PANAS demonstrated good internal consistency for both the positive (α = 0.949) and negative subscales (α = 0.881) and has demonstrated validity (Watson et al., 1988; Crawford and Henry, 2004).

### Data Analytic Approach

Analyses used linear mixed modeling in the Statistical Package for Social Sciences (SPSS v. 24.00). Generalized linear modeling (GLM) was chosen as an exploratory method of analysis, given the small study sample size and the fact that this is a pilot/feasibility study. This approach was also selected because it accounted for the non-independence of individual-level repeated measurements, thus allowing for maximal use of data from our small sample. Further, to adjust predictions given the small sample size, the Kenward-Roger method was implemented, which adjusts degrees of freedom for mixed effects models in small samples (Kenward and Roger, 1997). Finally, this approach was selected because GLM’s are generally considered to be robust to violations of assumptions. However, it is worth noting that due to the small sample size, assumptions such as homoskedasticity, normality, and linearity were not fully met for every model run.

For all outcome variables except the PANAS, timepoint (e.g., baseline) was specified as a fixed effect, and participants were specified as random effects. For PANAS analyses, “Session” refers to VR session number (1–13), while “Occasion” refers to either before or after the VR session. Both Session and Occasion were specified as fixed effects, and participants were specified as random effects. Analyses for the PANAS were first run as an interaction between Session and Occasion, and then run as main effects of Session and main effects of Occasion. Therefore, a total of 13 planned models were run.

## Results

Measurement means and standard deviations are reported in Supplementary Table 1.

### Outcome Measures

#### Anhedonia, Depression, Anxiety, and Functional Impairment

**FIGURE 1:**
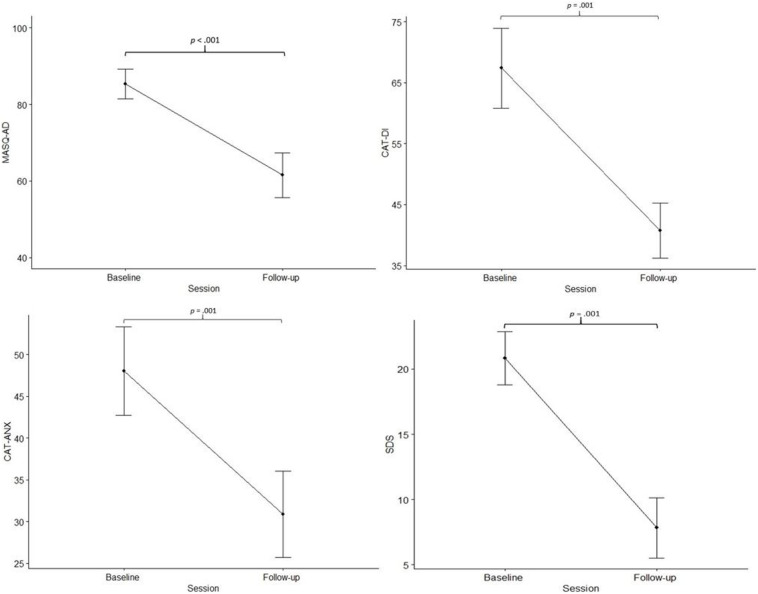
Measures of anhedonia, depression, and anxiety. Mean scores of item responses on the MASQ-AD, Mood and Anxiety Symptom Questionnaire-Anhedonic Depression; CAT-DI, Computerized Adaptive Testing-Depression; CAT-ANX, Computerized Adaptive Testing-Anxiety; and SDS, Sheehan Disability Scale; at baseline and 1-month follow-up. Error bars represent standard error of the mean.

MASQ-AD [*t*(17) = −4.875, *p* < 0.001], CAT-DI [*t*(17) = −3.892, *p* = 0.001], CAT-ANX [*t*(17) = −3.843, *p* = 0.001], and SDS significantly decreased from baseline to follow-up [*t*(17) = −6.347, *p* < 0.001] (Figure 1).

#### Reward Sensitivity

The BAS subscales did not change significantly from baseline to follow-up; Drive [*t*(17) = 0.594, *p* = 0.561], Fun Seeking [*t*(17) = 1.672, *p* = 0.113], and Reward [*t*(17) = 0.894, *p* = 0.384]. Neither the total score nor the TEPS subscales changed over time; Anticipatory subscale [*t*(17) = 1.032, *p* = 0.316], and Consummatory subscale [*t*(17) = 0.627, *p* = 0.539].

#### Positive and Negative Affect

**FIGURE 2:**
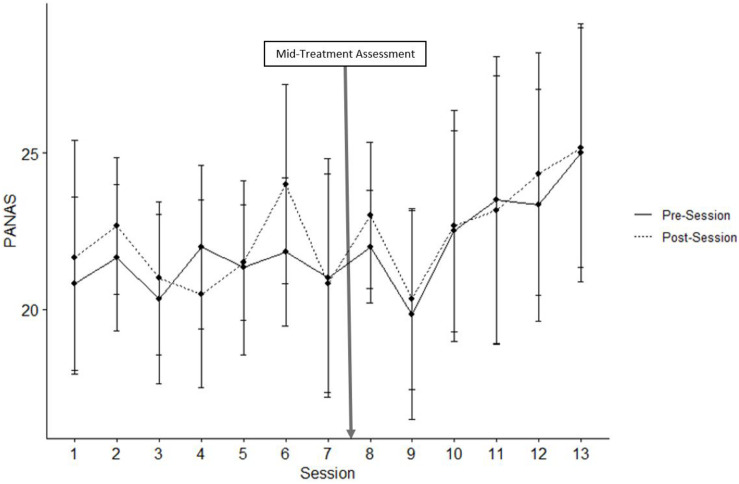
Positive PANAS. Mean scores of positive item responses on the PANAS before and after all thirteen VR sessions. Error bars represent standard error of the mean.

For PA across all 13 sessions, neither the interaction of Session × Occasion [*t*(125) = 0.420, *p* = 0.999], nor the main effects of Session [*t*(137) = 1.235, *p* = 0.122] or Occasion were significant [*t*(137) = 0.866, *p* = 0.388; see Figure 2]. Given visual inspection of the data, we conducted exploratory analyses of the first versus the second half of training. For PA in sessions 1–7, no effects were significant, *p*’s > 0.05. For sessions 8–13, PA increased significantly across Sessions [*t*(60) = 1.630, *p* = 0.031], although neither the interaction of Session × Occasion [*t*(55) = 0.0245, *p* = 0.997] nor the main effects of Occasion were significant [*t*(60) = 0.497, *p* = 0.621].

**FIGURE 3:**
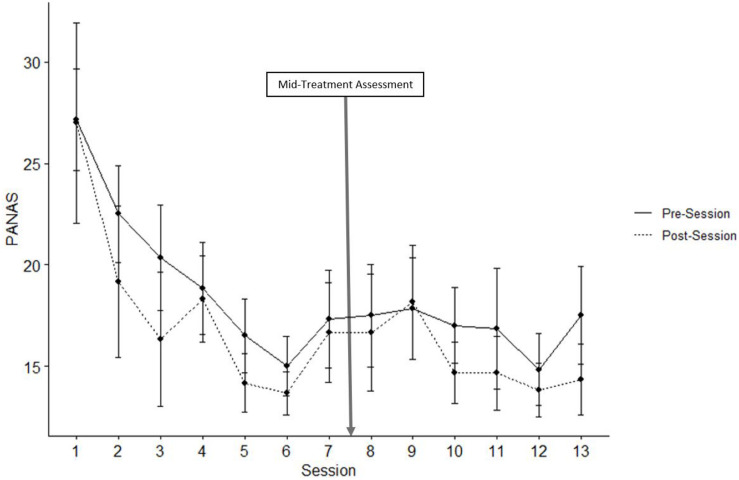
Negative PANAS. Mean scores of negative item responses on the PANAS before and after all thirteen VR sessions. Error bars represent standard error of the mean.

For NA across all 13 sessions, the main effect of Session was significant [*t*(137) = −2.402, *p* < 0.001] such that NA decreased across the 13 sessions, and the main effect of Occasion was significant [*t*(137) = −2.943, *p* = 0.004] such that NA decreased from before to after VR sessions (see Figure 3). The interaction of Session × Occasion was not significant [*t*(125) = 0.581, *p* = 0.980].

## Discussion

The purpose of this study was to investigate the effectiveness of positive VR scenes followed by imaginal recounting of the VR scene and of an autobiographic memory as a treatment for anhedonia in a sample of individuals with clinically significant depression. Our results suggest that the VR protocol significantly reduced symptoms of anhedonia, depression, anxiety and impairments in functioning. These changes coincided with significant reductions of negative affect throughout VR and with increases in positive affect throughout the last half of VR. The observed reductions in depression, anxiety, and functional impairment are similar to previous studies involving VR in anxious samples (Navarro-Haro et al., 2019). Decreases in anhedonia have also been reported in a case study of VR for PTSD (Gerardi et al., 2008), but to our knowledge, this is the first study to demonstrate the impact of positive VR scenes with imaginal recounting on anhedonia in the context of depression.

Our measures of reward sensitivity did not change over time. Trait dispositions toward reward may be unlikely to change over a 7-week treatment protocol. Future research should investigate measures of reward sensitivity that are more sensitive to treatment change. It is also conceivable that larger samples are needed to observe significant change on such trait measures.

Overall, the significant reduction in severity of self-report anhedonia symptoms from baseline to follow-up suggests the potential of VR with imaginal recounting as a treatment for anhedonia. We are not able to draw conclusions regarding the specific components of anhedonia (e.g., anticipation or motivation for reward, attainment of reward, learning of reward), but we speculate that this protocol primarily targets the subconstruct of initial response to reward attainment. Then, through repeated savoring of VR rewarding scenes, we hypothesize a downstream effect upon reward anticipation, which is consistent with evidence for state improvements in positive affect to increase motivation in daily life of individuals with anhedonia (Van Roekel et al., 2019).

There are several limitations to be noted. Since this was a pilot sample statistically constrained by its small size and lack of control group, our findings provide proof of concept alone and call for replication with larger samples and randomized controlled designs. More complex analyses suitable to a larger sample will also allow for the accounting of potential collinearity of outcome variables, which could qualify some of our results. Generalizability is also limited by our specific inclusion and exclusion criteria, which required participants to be moderately to severely depressed with low reward and high mental imagery skills. Furthermore, the simultaneous inclusion of VR scenes and imaginal recounting renders it impossible to parse the elements most accountable for therapeutic change. Overall, this study demonstrated the potential of positive experiences in VR as a novel and efficacious treatment for anhedonia symptoms and depression-related impairment.

## Data Availability Statement

The raw data supporting the conclusion of this article will be made available by the authors, without undue reservation.

## Ethics Statement

The studies involving human participants were reviewed and approved by the University of California, Los Angeles Medical Institutional Review Board 3 (IRB#16-001568). The participants provided their written informed consent to participate in this study.

## Author Contributions

All authors listed have made a substantial, direct and intellectual contribution to the work, and approved it for publication.

## Conflict of Interest

The authors declare that the research was conducted in the absence of any commercial or financial relationships that could be construed as a potential conflict of interest.

## Footnotes

1 Functional MRI was used to assess neural response to a monetary incentive delay task at baseline, mid-treatment and post-treatment but results are not presented as they are outside the scope of this brief report.
