# Supplementary material for: Virtual Reality Reward Training for Anhedonia: A Pilot Study
Source: Front Psychol. 2021 Jan 7;11:613617. doi: 10.3389/fpsyg.2020.613617 (PMC7817899; doi:10.3389/fpsyg.2020.613617)
Supplement: Supplementary file 1 [file Table_1.DOCX]

Supplementary Material

# Supplementary Table

| **Supplementary Table 1** | | |  | |  | |  | |
| --- | --- | --- | --- | --- | --- | --- | --- | --- |
| *Outcome Measure Means and Standard Deviations* | | | | | | |  | |
| **Outcome Measures** | **Baseline** | | **Mid-Treatment** | | **Post-Treatment** | | **Follow-Up** | |
|  | *M* | *SD* | *M* | *SD* | *M* | *SD* | *M* | *SD* |
| *Symptoms of Depression, Anxiety, and Anhedonia* | | | |  |  |  |  |  |
| **CAT-DI** | 67.38 | 16.00 | 52.52 | 12.95 | 49.92 | 12.73 | 40.77 | 11.03 |
| **CAT-ANX** | 48.00 | 12.97 | 40.55 | 20.40 | 33.78 | 7.84 | 30.90 | 12.62 |
| **MASQ-AD** | 85.33 | 9.44 | 72.00 | 11.10 | 66.67 | 11.17 | 61.50 | 14.35 |
| **SDS** | 20.83 | 4.96 | 13.83 | 7.57 | 10.50 | 5.68 | 7.83 | 5.64 |
| *Reward Sensitivity* |  |  |  |  |  |  |  |  |
| **BAS – Drive** | 8.83 | 2.79 | 8.83 | 1.94 | 9.33 | 1.75 | 9.17 | 1.47 |
| **BAS – Fun Seeking** | 11.00 | 2.61 | 11.00 | 1.26 | 11.50 | 1.87 | 12.17 | 1.94 |
| **BAS – Reward** | 15.00 | 2.45 | 14.00 | 1.10 | 15.17 | 1.17 | 15.50 | 2.43 |
| **BAS - Total** | 34.83 | 7.14 | 33.83 | 2.48 | 36.00 | 2.19 | 36.83 | 2.86 |
| **TEPS – Anticipatory** | 35.00 | 5.93 | 36.33 | 5.05 | 38.00 | 6.23 | 36.67 | 8.62 |
| **TEPS - Consummatory** | 37.00 | 3.90 | 38.17 | 4.88 | 37.67 | 5.96 | 38.17 | 7.14 |
| **TEPS - Total** | 72.00 | 6.39 | 74.50 | 7.89 | 75.67 | 11.15 | 74.83 | 15.20 |
| *Note.* CAT-DI: Computerized Adaptive Testing-Depression, CAT-ANX: Computerized Adaptive Testing-Anxiety, MASQ-AD: Mood and Anxiety Symptom Questionnaire-Anhedonic Depression, SDS: Sheehan Disability Scale, BAS: Behavioral Activation Scale, TEPS: Temporal Experience of Pleasure Scale. | | | | | | | | |

# Supplementary Figure


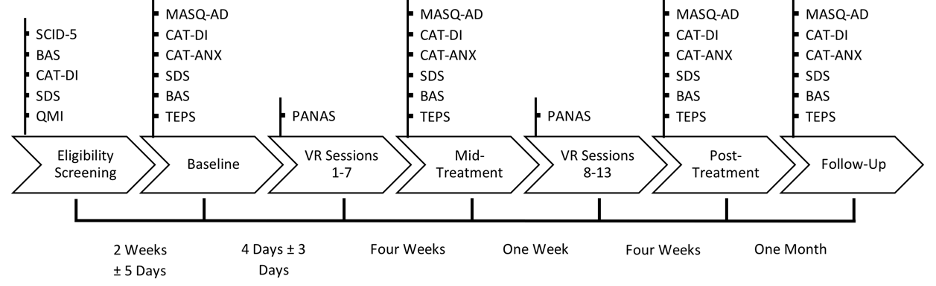


*Supplementary Figure 1.* Timeline of Assessments. CAT-ANX: Computerized Adaptive Testing-Anxiety, CAT-DI: Computerized Adaptive Testing-Depression, MASQ-AD: Mood and Anxiety Symptom Questionnaire-Anhedonic Depression, PANAS: Positive and Negative Affect Schedule, QMI: Questionnaire Upon Mental Imagery, SCID-5: Structured Clinical Interview for the DSM-5, SDS: Sheehan Disability Scale, BAS: Behavioral Activation Scale, TEPS: Temporal Experience of Pleasure Scale.
